# The Comparability of Anti-SARS-CoV-2 IgG Levels Measured with Different Immunoassays Varies over Time after Primary BNT162b2 Vaccination and Homologous Booster Immunization

**DOI:** 10.1128/spectrum.03022-22

**Published:** 2022-11-10

**Authors:** Gian Luca Salvagno, Giuseppe Lippi

**Affiliations:** a Section of Clinical Biochemistry, University of Verona, Verona, Italy; b Service of Laboratory Medicine, Pederzoli Hospital, Peschiera del Garda, Italy; University of Guelph

**Keywords:** antibodies, booster, COVID-19, SARS-CoV-2, immunization

## LETTER

In a recent article published in this journal, Perkmann and colleagues concluded that the interassay comparability of anti-Spike SARS-CoV-2 (severe acute respiratory syndrome coronavirus 2) antibodies may be substantially time dependent (1). To provide further insights on this important aspect, and considering also that Perkmann et al. evaluated different antibody classes in their work (i.e., total antibodies versus IgG), we compared the results of two anti-SARS-CoV-2 IgG tests at different time points in subjects receiving primary BNT162b2 vaccination, extending such comparison also after homologous booster immunization.

This serosurveillance study was based on a cohort of ostensibly healthy workers of Pederzoli Hospital (Peschiera del Garda, Verona, Italy), who voluntarily received primary coronavirus disease 2019 (COVID-19) vaccination with Pfizer/BioNTech BNT162b2 (Pfizer Inc., New York, USA; double 30-μg dose with 3-weeks interval), followed by homologous booster (30-μg) immunization. Whole blood was collected throughout the study at different time points, as summarized in Table 1. Serum was separated and used for testing anti-SARS-CoV-2 IgG antibodies using two different tests, i.e., DiaSorin Trimeric spike IgG immunoassay (DiaSorin, Saluggia, Italy) and Maglumi SARS-CoV-2 S-RBD IgG (New Industries Biomedical Engineering Co., Ltd. [Snibe], Shenzhen, China). Results were compared with Spearman’s correlation and Bland and Altman plots, using Analyse-it (Analyse-it Software Ltd., Leeds, UK). All participants signed an informed consent for participating in the study. The study was conducted in accordance with the Declaration of Helsinki and approved by the Ethics Committee of Verona and Rovigo Provinces (59COVIDCESC; 3 November 2021).

**TABLE 1:** Spearman’s correlation and bias expressed in ratio between the values of anti-SARS-CoV-2 antibodies measured with DiaSorin Trimeric spike IgG and those measured by Maglumi SARS-CoV-2 S-RBD IgG

Timing	Correlation (95% CI^*a*^)	Bias in ratio (95% CI)
21 days after dose 1	0.86 (0.79–0.91; *P* < 0.001)	2.5 (2.3–2.8)
1 mo after dose 2	0.82 (0.73–0.89; *P* < 0.001)	3.8 (−0.9–8.6)
3 mo after dose 2	0.86 (0.79–0.91; *P* < 0.001)	5.6 (5.1–6.1)
6 mo after dose 2	0.83 (0.74–0.89; *P* < 0.001)	8.1 (7.4–8.8)
9 mo after dose 2	0.81 (0.71–0.88; *P* < 0.001)	10.5 (9.1–11.8)
1 mo after dose 3	0.89 (0.83–0.93; *P* < 0.001)	6.3 (5.4–7.1)

a 95% CI, 95% confidence interval.

The final study population consisted of 74 health care workers (43 ± 12 years, 41 females). The main results are summarized in Table 1 (and in the supplemental material). Spearman’s correlation between the two immunoassays remained always considerably high, ranging between 0.81 and 0.89, reaching the highest value 1 month after booster immunization. The values (in both cases reported in binding antibody units per milliliter) of DiaSorin Trimeric spike IgG were consistently higher than those obtained with Maglumi SARS-CoV-2 S-RBD IgG, and the ratio between these tests gradually increased over time from the administration of the first vaccine dose up to 9 months after the second dose (i.e., the ratio increased from 2.5 to 10.5). A different trend was then noted 1 month after booster immunization, with the bias between these tests decreasing to 6.3 (Table 1).

In conclusion, these results confirm that the comparability of anti-Spike SARS-CoV-2 antibody tests, even within the same antibody class (i.e., IgG), seems to be always high, but the bias of test results varies widely depending on timing of sample collection after primary and even after booster immunization.
