# Supplementary material for: The Comparability of Anti-SARS-CoV-2 IgG Levels Measured with Different Immunoassays Varies over Time after Primary BNT162b2 Vaccination and Homologous Booster Immunization
Source: Microbiol Spectr. 2022 Nov 10;10(6):e03022-22. doi: 10.1128/spectrum.03022-22 (PMC9769787; doi:10.1128/spectrum.03022-22)

SUPPLEMENTAL FILE 1. Correlation between the values of anti-SARS-CoV-2 antibodies measured with DiaSorin Trimeric spike IgG vs. MAGLUMI SARS-CoV-2 S-RBD IgG.

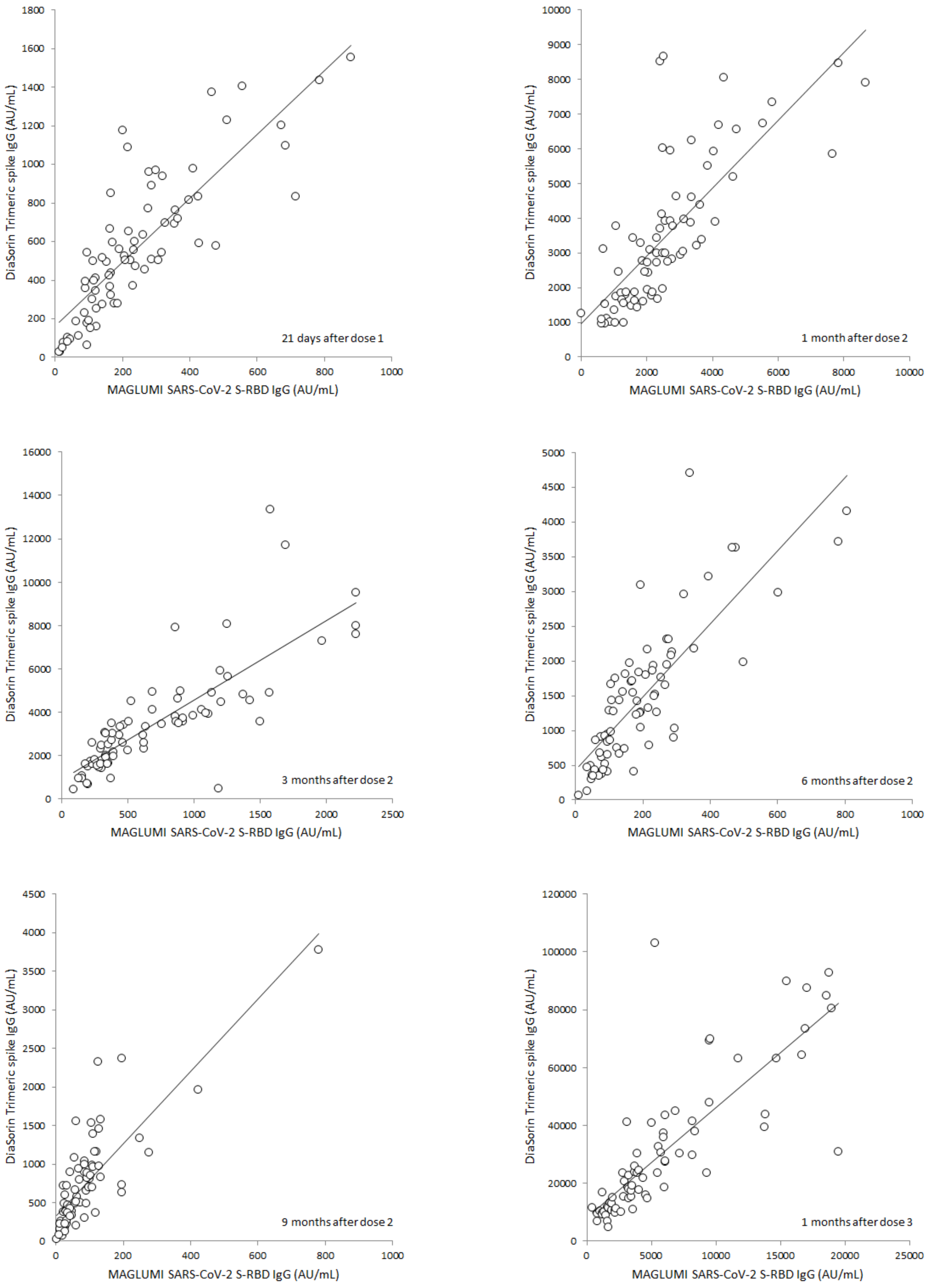

Supplement: Supplemental file 1 — Supplemental material. Download spectrum.03022-22-s0001.pdf, PDF file, 0.2 MB [file spectrum.03022-22-s0001.pdf]
